# Untargeted Metabolomic Profiling Reveals Variation in Metabolites Associated with Nutritional Values in Tef Accessions

**DOI:** 10.1007/s11130-021-00931-6

**Published:** 2021-11-11

**Authors:** Aiswarya Girija, Rattan Yadav, Fiona Corke, John Doonan, Luis A. J. Mur

**Affiliations:** Institute of Biological, Environmental and Rural Studies (IBERS), Aberystwyth University, Aberystwyth, SY23 3DA Ceredigion UK

**Keywords:** Tef, Gluten-free, Untargeted metabolite profiling, Flavonoid, Vitamins

## Abstract

Tef (*Eragrostis tef*), is a gluten-free orphan cereal, crop of nutritional and economical significance. Here we used untargeted metabolomics to survey metabolite variation in 14 diverse tef accessions at 15-days post germination. Tef genotypes were classified into four metabolomic groups where variation was linked to flavones and flavonols. Further analysis on white seeded accessions shows variation related to sucrose and important vitamins, nicotinamides (vitamin B3) riboflavin (vitamin B2) and folate (vitamin B9). Coloured seeded accessions showed variation in metabolism related to amino acid and sugars. This study highlights the potential of metabolomics in exploring the nutritional traits in tef.

**Supplementary Information:**

The online version contains supplementary material available at 10.1007/s11130-021-00931-6.

One of the main challenges to future food security is feeding an increasing population with healthy and nutritional foods. So, researchers are now exploring new “orphan” crops to meet daily dietary requirements. One such orphan crop is an Ethiopian cereal, Tef (*Eragrostis tef*) that is gaining popularity due to its gluten-free and highly nutritious grain [1]. Despites its nutritional value, tef is still an understudied crop due to low investment in research and breeding [2]. Metabolomics can reveal information on agronomic and nutritional traits in crops [3] but has yet to be applied to tef. We report the first untargeted metabolite profiling of tef seedlings to facilitate the rapid identification of phytonutrients in varied accessions.

The 14 diverse tef accessions were obtained from NPGS-GRIN database, https://npgsweb.ars-grin.gov/gringlobal/search (Table.S1). Plants were germinated in Levington F2 compost and grown under controlled environmental conditions at 24/21 °C ± 2 °C, 14/10 h day/night photoperiod. 15-day old tef seedlings were harvested and ~ 40 mg (fresh shoot weight) was used for metabolomic assessment as described Skalska et al. [4]. All data generated or analysed during this study are included in supplementary information files.

Seedlings were harvested for metabolomic assessment at 15-days did not exhibit any obvious morphological variation (Fig. S1). Initial principal component analysis (PCA) (Fig. S2a) and dendrograms (Fig. S2b) suggested four broad groups of accessions. Metabolite variation within tef genotypes did not reflect geographical origin or morphology. Indeed, flavone and flavonols were found to be the major sources of variation within the tef genotypes (Table.S2, Fig. S3a, S3b) and other enhanced pathways were starch and sucrose, panthothenate (vitamin B5), CoA, thiamine (vitamin B1) and anthocyanin metabolism. To reduce the genotypic diversity under examination, we undertook separate metabolite analysis for white (Fig. 1a, b) and coloured (Fig. 1c, d). Two interesting metabolites; kaempferol and sorbitol with possible links to seed colour were observed, the former was high in coloured whereas latter was high in white accessions (Fig.S4a, S4b). Within the white accessions, Ada, Alba and Manyi were distinct from all other accessions which clustered together (Fig. 1a). Enrichment of significant metabolites identified features that are relevant to human nutrition; sucrose, and vitamins; nicotinamides (vitamin B3) riboflavin (vitamin B2) and folate (vitamin B9) (Fig. 1b). The metabolite variation within coloured accessions also showed genotypic differences, Gea-lamie and Dabbi formed one group whereas Karadebi and Red dabi were together (Fig. 1c), with pathway enrichment showing a bias towards amino acid metabolism (Fig. 1d). The distinct metabolite variations in white (Fig. 2a) and coloured seed (Fig. 2b) accessions are indicated by heat maps.

**Fig.1:**
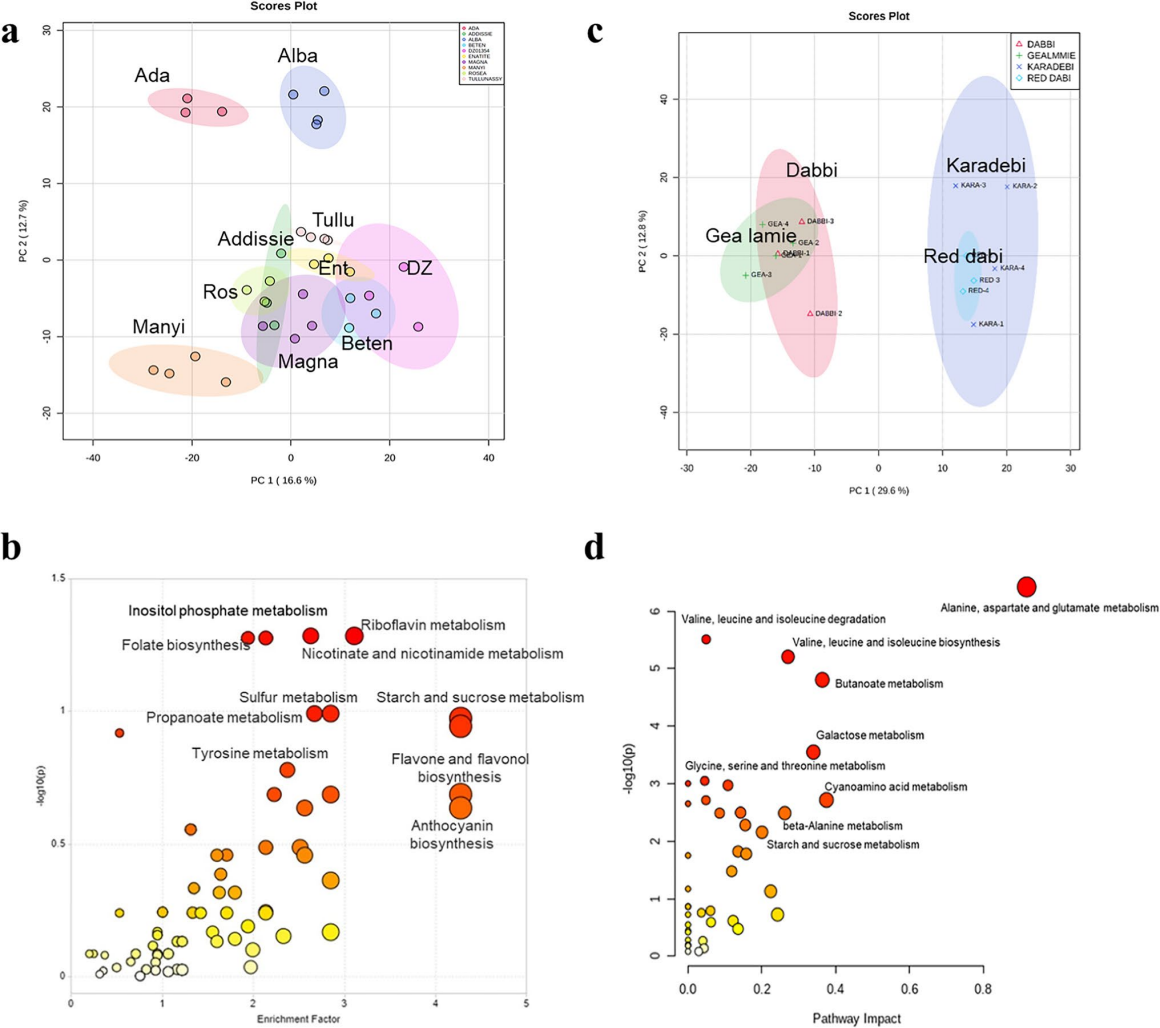
Principal component analysis (PCA) and functional enrichment analysis of white (**a**), (**b**) and coloured (**c**), (**d**) tef accessions

**Fig. 2:**
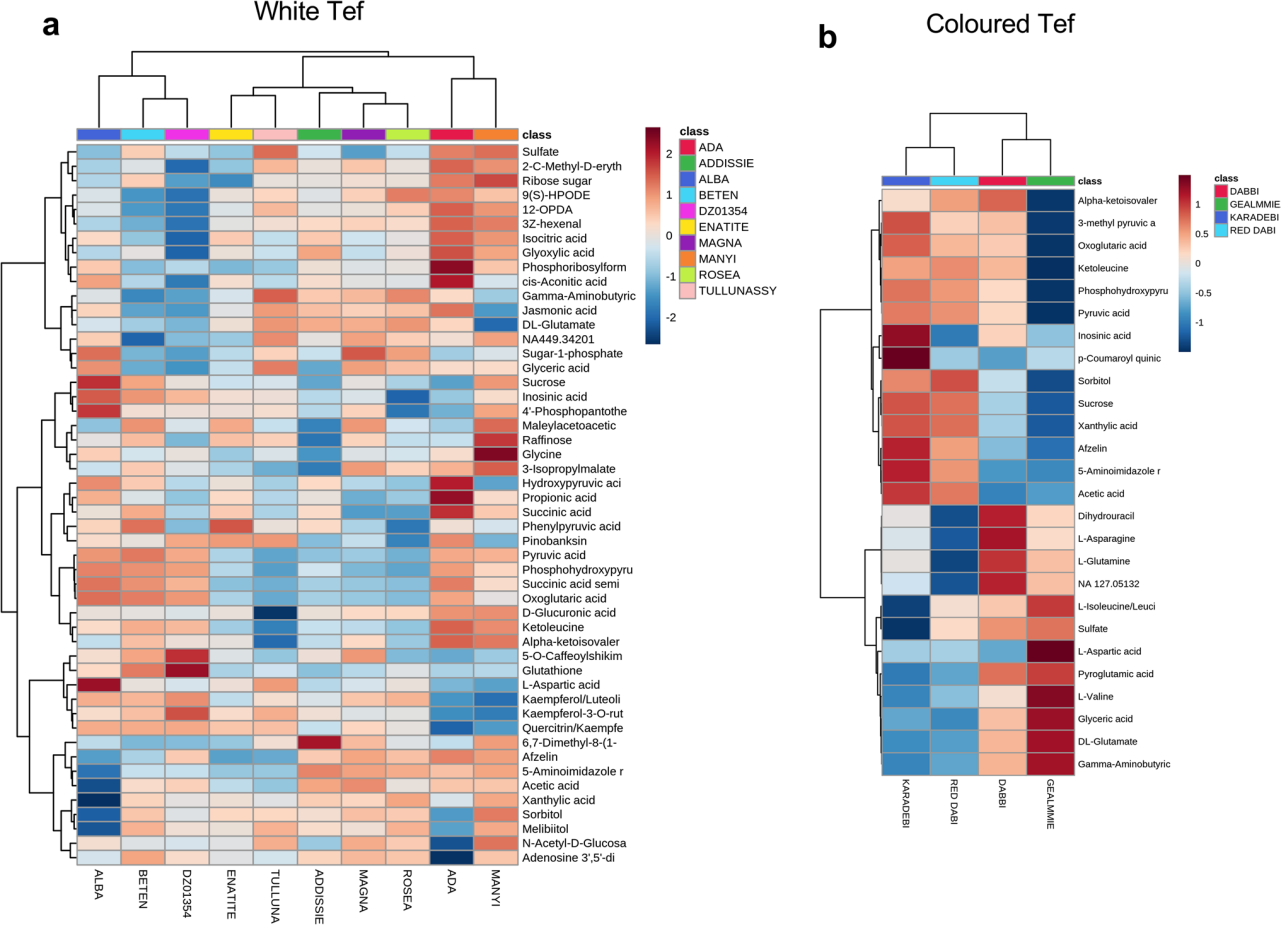
(**a**) Heat map showing distribution of 50 identified and annotated metabolites within white seed tef genotypes (**b**) top 26 metabolites within coloured seed genotypes

Improving understudied crops like tef could offer an alternative source of human food and animal feed. In this study, we show the applicability of untargeted metabolomics in elucidating the regulatory networks in tef related to nutrition.

From our data, we can conclude that at the early seedling stage tef genotypes exhibited metabolite variation which was related to nutritional richness [5]. Besides tef seedlings being potentially used as functional foods (microgreens) for human consumption, metabolomic screening of such seedlings could ease the rapid targeting of key progeny in a segregating breeding population.

## Supplementary Information

Below is the link to the electronic supplementary material.

Supplementary file1 (DOCX 1024 KB)

## Data Availability

The authors declare that the data supporting the findings of this study are available within the article [and its supplementary information files].
