# Supplementary material for: Untargeted Metabolomic Profiling Reveals Variation in Metabolites Associated with Nutritional Values in Tef Accessions
Source: Plant Foods Hum Nutr. 2021 Nov 11;76(4):536–9. doi: 10.1007/s11130-021-00931-6 (PMC8629858; doi:10.1007/s11130-021-00931-6)

**Supplementary data**

**Table.S1: Details of teff accessions used for the study**. Teff seeds were obtained from NPGS-GRIN germplasm. Table represents the accession details, name, region of origin, seed color, plant height, panicle type., group. Each genotype is assigned a group number from 1-4 based on tentatively identified metabotypes.

| **Serial No:** | **Accession number** | **Genotype/**  **Cultivar name** | **Area/Altitude** | **Seed colour** | **Plant height** | **Panicle type** | **Groups** |
| --- | --- | --- | --- | --- | --- | --- | --- |
| 1 | PI 524438 | Dabbi | Gojam, Ethiopia | red | 55-58 cm | very loose | 2 |
| 2 | PI 524440 | Gea-lamie | Wellega, Ethiopia | red | 20-50 cm | very loose | 2 |
| 3 | PI 524442 | Karadebi | Wellega, Ethiopia | red | 40-80 cm | very loose | 4 |
| 4 | PI 557457 | Red dabi | Ethiopia | brown | 91 cm | very loose | 4 |
| 5 | PI 524433 | Ada | Shewa, Ethiopia | white | 50-100 cm | semi compact | 1 |
| 6 | PI 524434 | Addisie | Shewa, Ethiopia | white | 65-90 cm | very compact | 2 |
| 7 | PI 524435 | Alba | Shewa,Ethiopia /1700-2500m | white | 60-135 cm | fairly loose | 4 |
| 8 | PI 524437 | Beten | Shewa, Ethiopia | white | 40-85 cm | very loose | 4 |
| 9 | PI 524439 | Enatite | Shewa,Ethiopia/2000m | white | 45-85 cm | very loose | 4 |
| 10 | P1 524443 | Manyi | Shewa, Ethiopia | white | 40-100 cm | fairly loose | 3 |
| 11 | PI 524444 | Rosea | Shewa, Ethiopia | white | 50-95 cm | fairly loose | 4 |
| 12 | PI 524445 | Tullu Nasy | Wellega, Ethiopia | white | 30-50 cm | very loose | 4 |
| 13 | PI 557456 | DZ-01-354 | Ethiopia | white | 125 cm | fairly loose | 4 |
| 14 | PI 243908 | Magna | Ethiopia | white | 110 cm | fairly loose | 4 |

**Table S2:** Significant metabolite features associated with flavone and flavonol biosynthesis within 14 teff genotypes. Query mass denotes the masses identified from the untargeted metabolomics datasets. The annotations are made based on the rice reference metabolome, masses, compound ID’s and formula are derived from KEGG library

| No | Query.Mass | Matched.  Compound (KEGG ID) | Matched.  Form | Hit | Exact mass (KEGG) | Mol.  weight (KEGG) | Formula (KEGG) |
| --- | --- | --- | --- | --- | --- | --- | --- |
| 1 | 285.04156 | C05903/  C01477/  C01514 | M-H[-] | Kaempferol/Luteolin/Apigenin | 286.0477 | 286.2363 | C15H10O6 |
| 2 | 413.08948 | C16911 | M-H2O-H[-] | Afzelin | 432.1056 | 432.3775 | C21H20O10 |
| 3 | 429.08463 | C01750/  C12249 | M-H2O-H[-] | Quercitrin/Kaempferol-3-O-glucoside | 448.1006 | 448.3769 | C21H20O11 |
| 4 | 431.10034 | C16911 | M-H[-] | Quercetin 3-glucoside | 432.1056 | 432.3775 | C21H20O10 |
| 5 | 432.10315 | C16911 | M(C13)-H[-] | Afzelin | 432.1056 | 432.3775 | C21H20O10 |
| 6 | 447.09537 | C01750/  C12249 | M-H[-] | Kaempferol 3-O-rhamnoside-7-O-glucoside/Kaempferol-3-O-glucoside | 448.1006 | 448.3769 | C21H20O11 |
| 7 | 448.09863 | C01750/  C12249 | M(C13)-H[-] | Quercitrin/Kaempferol-3-O-glucoside | 448.1006 | 448.3769 | C21H20O11 |
| 8 | 485.06958 | C05623 | M+Na-2H[-] | Quercetin 3-O-glucoside | 464.0955 | 464.3763 | C21H20O12 |
| 9 | 513.02112 | C16911 | M+Br81[-] | Afzelin | 432.1056 | 432.3775 | C21H20O10 |
| 10 | 523.10883 | C05623 | M+CH3COO[-] | Quercetin 3-O-glucoside | 464.0955 | 464.3763 | C21H20O12 |
| 11 | 593.15399 | C21833/  C21854 | M-H[-] | Kaempferol-3-O-rutinoside/Kaempferol 3-O-rhamnoside-7-O-glucoside | 594.1585 | 594.5181 | C27H30O15 |
| 12 | 647.11829 | C05625/  C19796 | M+Cl37[-] | Rutin/Quercetin 3-O-rhamnoside 7-O-glucoside | 610.1534 | 610.5175 | C27H30O16 |

**Figure.S1: Fresh shoot weight of teff seedlings**. 15- day old Teff seedlings (14 genotypes/accessions) harvested and used for metabolite extraction. The data represented as average fresh weight (mg) ± standard deviation (SD) of three to four biological replicates. Independent seedling is taken as each biological replicate. Statistically significant changes (Tukey- Kramer HSD test, p ≤ 0.05) between genotypes are identified by different letters.

**
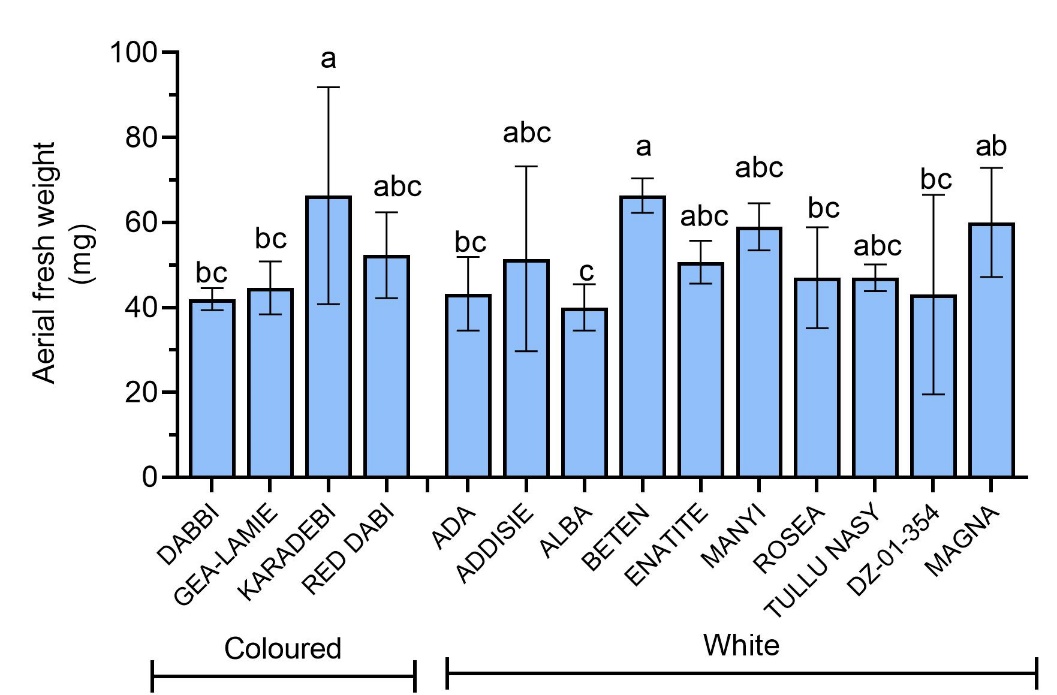
**

**Figure S2. Initial mapping of teff seedling metabolome (a)** Principal component analysis **(b)** Dendrogram showing clustering of 14 teff genotypes based on the metabolite features. Four groups, ‘metabotypes’ were identified based on the m/z features on negative mode.


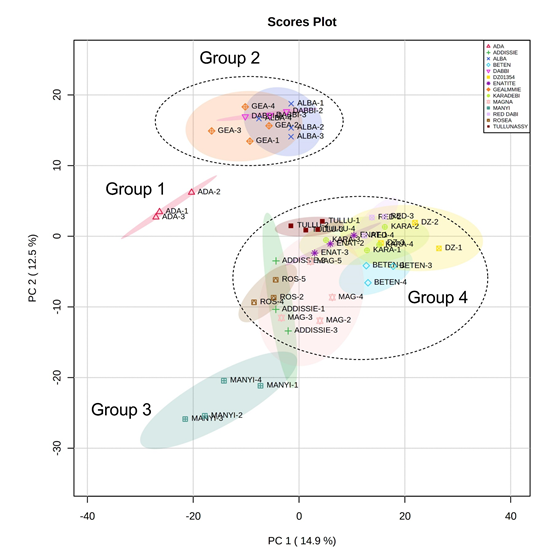


**a**


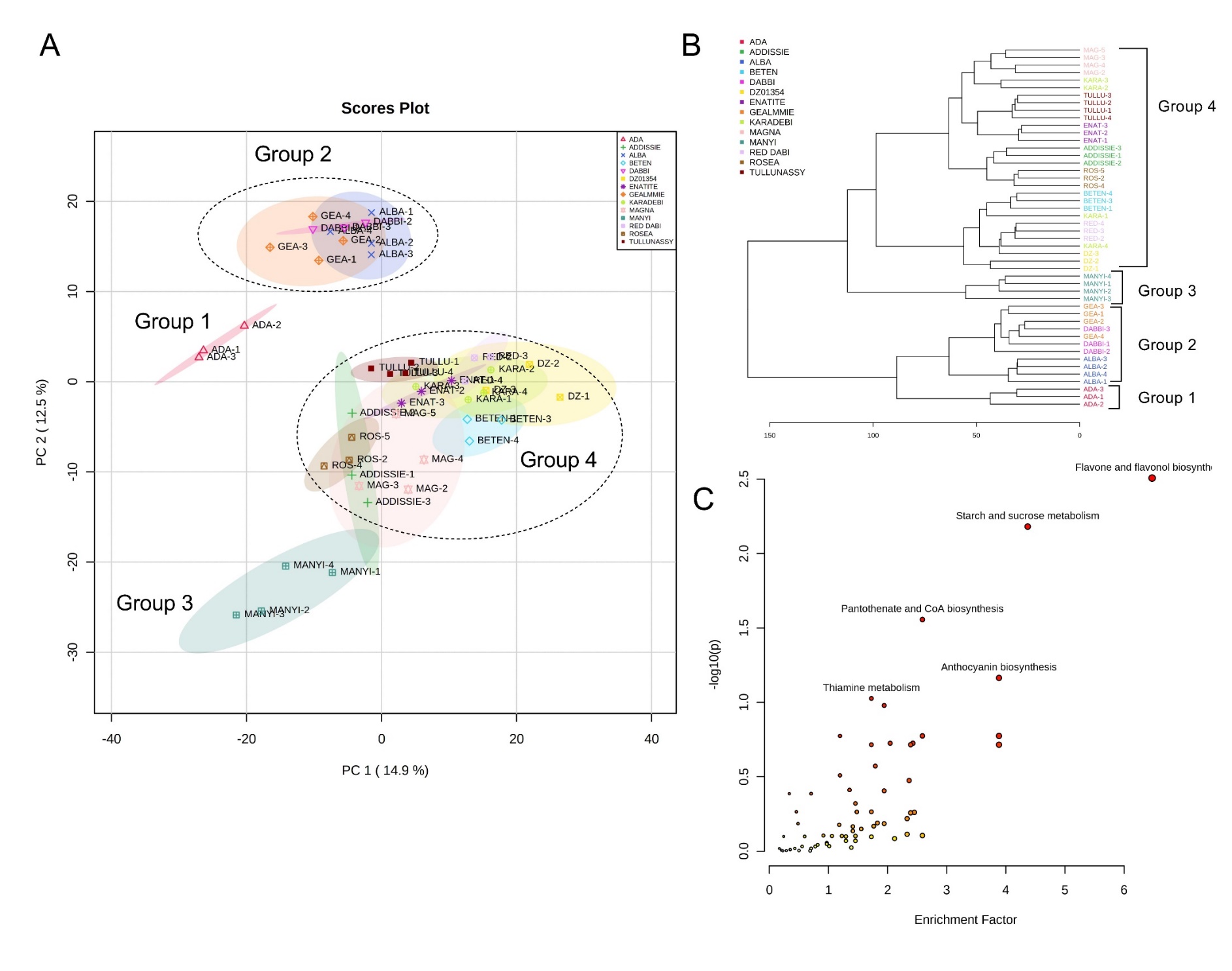


**b**

**
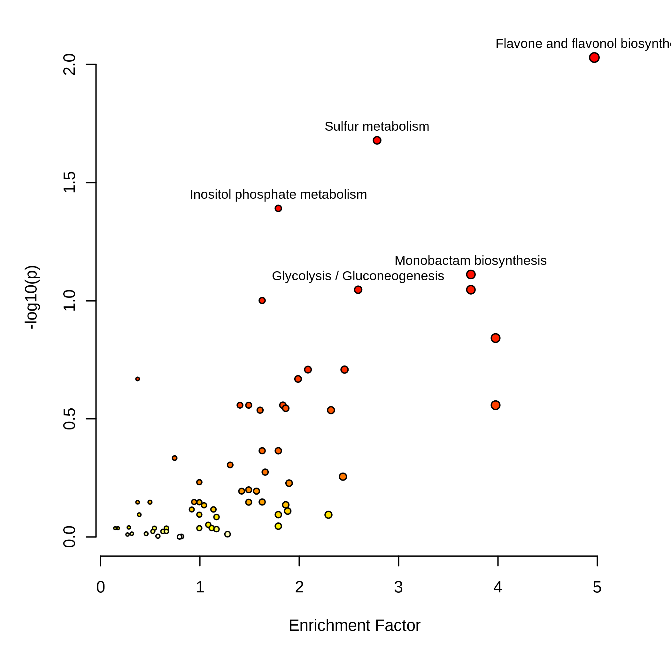
Figure S3. (a)** Pathway enrichment analysis of metabolites **(b)**Heat map showing distribution of metabolites identified from flavone and flavonol biosynthesis within the teff genotypes. Seed groups are white = w and coloured (red/brown) = C. Group refers to the metabolomic group defined in Figure 1.

**a**

**b**

**
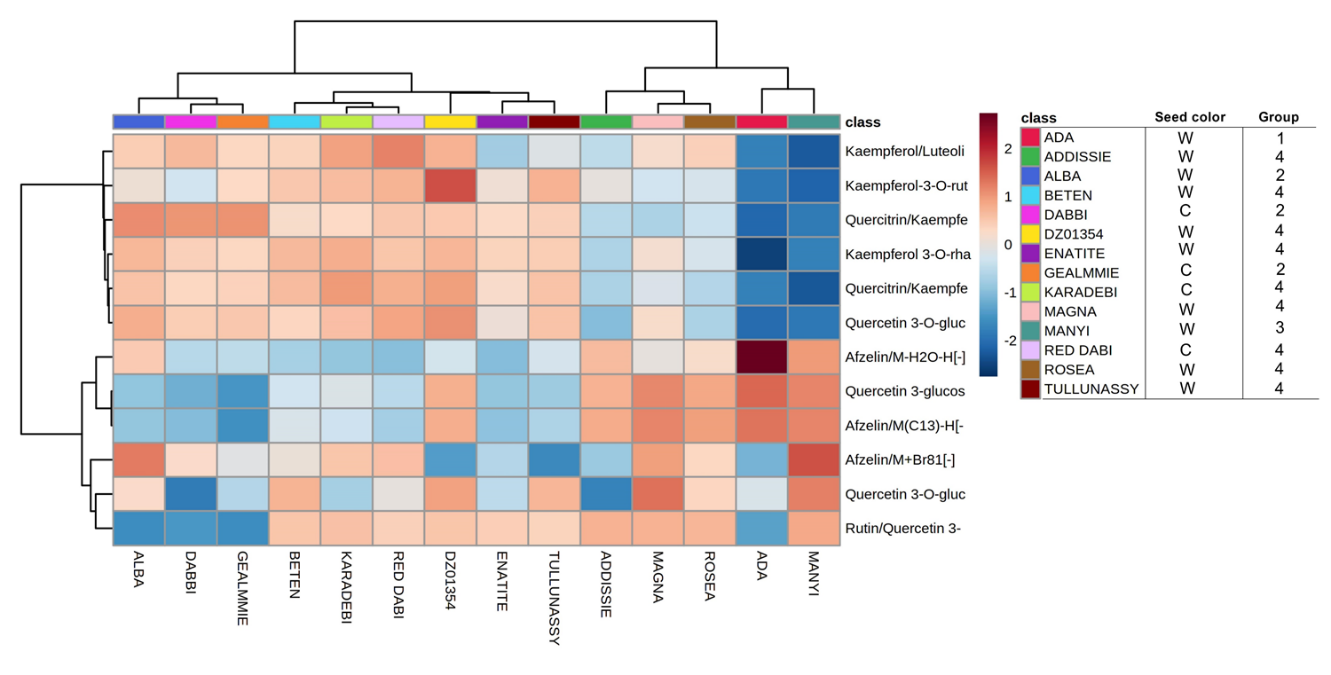
**

**Fig S4**: **(a)** PCA showing the metabolite variation between white and coloured seed accessions. **(b)** Heatmap showing 10 annotated significant metabolites within white and colored accessions


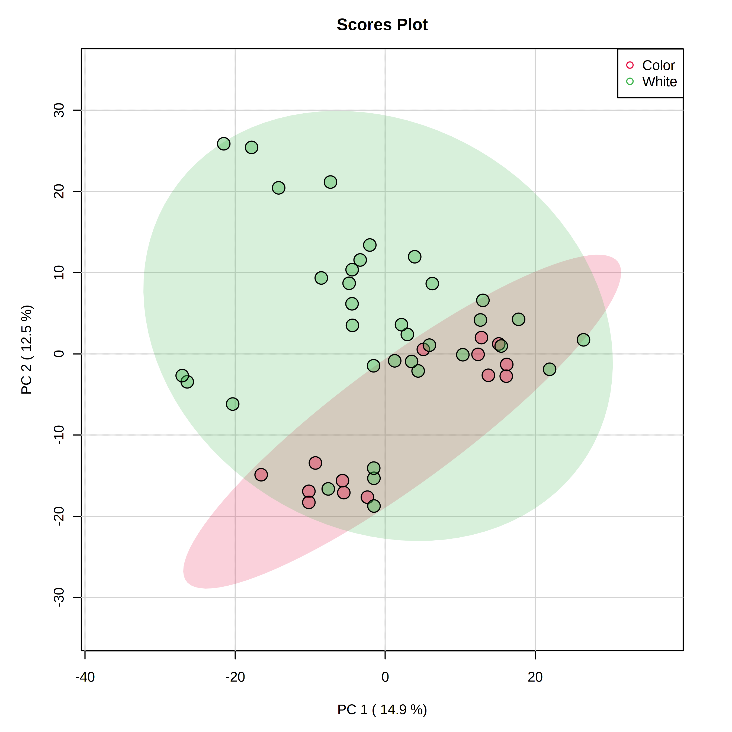

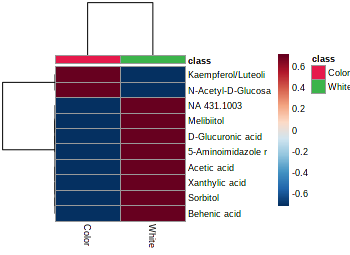

Supplement: Supplementary file 1 — Supplementary file1 (DOCX 1024 KB) [file 11130_2021_931_MOESM1_ESM.docx]
